# Correlation between Doppler, Manual Morphometry, and Histopathology Based Morphometry of Radial Artery as a Conduit in Coronary Artery Bypass Grafting

**DOI:** 10.1155/2016/8047340

**Published:** 2016-03-07

**Authors:** Om Prakash Yadava, Vinod Sharma, Arvind Prakash, Vikas Ahlawat, Anirban Kundu, Bikram K. Mohanty, Rekha Mishra, Amit K. Dinda

**Affiliations:** ^1^Department of Cardiothoracic Surgery, National Heart Institute, 49-50 Community Centre, East of Kailash, New Delhi 110065, India; ^2^Department of Cardiology, National Heart Institute, 49-50 Community Centre, East of Kailash, New Delhi 110065, India; ^3^Department of Cardiac Anesthesiology, National Heart Institute, 49-50 Community Centre, East of Kailash, New Delhi 110065, India; ^4^Department of Pathology, All India Institute of Medical Sciences, New Delhi 110029, India

## Abstract

*Background*. Long-term graft patency is the major factor impacting survival after coronary artery bypass grafting. Arteries are superior in this regard. Radial artery is considered the second best conduit after internal mammary artery. Several studies have shown excellent radial artery patency. We evaluated the morphologic characteristics of radial artery by three modalities, (i) preoperative Doppler ultrasound, (ii) intraoperative manual morphometry, and (iii) postoperative histology-based morphometry, and compared these with the aim of validating Doppler as a noninvasive test of choice for preoperative assessment of radial artery.* Methods*. This was a prospective study involving 100 patients undergoing coronary artery bypass grafting in which radial artery was used. The radial artery was assessed using preoperative Doppler ultrasound studies, intraoperative morphometry, and postoperative histopathology and morphometry. The morphometric measurements included (i) luminal diameter, (ii) intimal and medial thickness, and (iii) intima-media thickness ratio.* Results*. Using Bland-Altman plots, there was a 95% limit of agreement between the preoperative Doppler measurements and the postoperative histopathology and morphometry.* Conclusion*. Doppler ultrasound is an accurate screening test for evaluation of radial artery, in terms of intimal/medial thickness and luminal diameter as a conduit in coronary artery bypass grafting and has been validated by both morphometric and histopathology based studies.

## 1. Introduction

Long-term bypass graft patency is the major factor impacting survival in patients after coronary artery bypass grafting (CABG). It has been proved that arterial grafts are superior to venous grafts in terms of long-term patency. Among the arterial grafts, radial artery (RA) is considered to be the best conduit next to internal mammary artery. Use of RA has been supported by the result of several angiographic studies that have shown excellent short-, medium-, and long-term patency rates. A good conduit has to be of good caliber and should be free from pathological wall thickening. In this study, we have evaluated the morphologic characteristics of RA like luminal diameter and wall thickness by three different modalities, namely, (i) preoperative Doppler ultrasound (USG), (ii) intraoperative manual morphometry, and (iii) postoperative histology-based morphometry, and have compared the measurements of these three modalities with the aim of validating Doppler as a noninvasive test of choice for preoperative measurements of RA morphology.

## 2. Patients and Methods

This prospective study included 100 patients undergoing CABG between September 2012 and February 2013 at the National Heart Institute, New Delhi, in whom RA was used as a conduit. The study was approved by the institutional Ethics Committee and informed written consent was obtained from all patients prior to start of the study. The suitability of RA was assessed by modified Allen's test in the nondominant forearm at the bedside using pulse oximeter. All the patients who had a negative modified Allen's test, which signified a complete palmar arch, were subjected to Doppler USG of RA, which was performed by a single experienced observer using PHILIPS–IE-33 with 7.5 MHz Phased Array Rectangular Vascular Probe. Thorough and complete scanning of the RA was done starting just after ulnar artery branching (proximally) up to the wrist (distally). The scanning evaluation included (i) luminal diameter, (ii) measurement of intimal thickness and medial thickness, and (iii) measurement of intima-media thickness ratio.

In the operating room, after the patient was anaesthetized, the RA was harvested with its pedicle using an open, no-touch technique simultaneously with sternotomy and harvesting of other conduits. Monopolar diathermy and clips were used in dissection. After the RA was exposed, visual assessment for any gross abnormality was made, along with diameter of the vessel. RA was palpated for assessment of wall thickness and calcification. After the RA was harvested, sections of 1 cm length from both ends were cut with fine scissors before hydrostatic dilatation and storage with heparinized saline. No vasodilator fluid was used for storage. The luminal diameter and thickness of arterial wall were measured using a Vernier caliper. After intraoperative morphometry measurements, specimens constituting the proximal and distal ends were preserved in 5% formaldehyde solution and sent for histopathology study. 5–20 sections were analyzed per segment of artery submitted for evaluation. These were cross-sectioned at 5 micrometers and stained with hematoxylin-eosin, Verhoef van Gieson's elastic stain, and Masson's Trichrome Stain. Histopathological assessments were followed by evaluation of the slides by another pathologist having expertise in morphometric measurements, who was blinded to the previous findings. The specimens were analyzed with a color image analysis system. The morphometric measurements included (i) luminal diameter, (ii) intimal and medial thickness, and (iii) intima-media thickness ratio. Any fibromyointimal proliferation between the endothelium and internal elastic lamina was considered as indicating intimal hyperplasia. An atherosclerotic lesion was defined by intimal lipid lying free as cholesterol clefts or in aggregates of foamy macrophages. Medial calcification was recorded if present.

### 2.1. Statistical Analysis

Data was assessed and represented in mean values and association in categorical variables was evaluated by Fisher's exact/chi-square test. In case of continuous variables, two groups were compared by using *t*-test. Agreement in the two methods for the continuous variables was seen by Bland Altman Plot. Intraclass correlation was calculated with 95% confidence interval.

## 3. Observations and Results

### 3.1. Patient Demography

One hundred patients, who fulfilled the inclusion criteria of use of RA (on the basis of modified Allen's test) as a conduit in CABG, were included in this prospective study. Two patients were excluded as preoperative Doppler showed extensive calcification in one and luminal diameter <2 mm in another. There was no age bar. The minimum age of the patients was 44 years and the maximum age was 80 years, the mean being 61.45 years. 79 patients were males and 21 patients were females. The patient demographics are shown in Table 1.

### 3.2. Preoperative Radial Doppler Measurements

The mean luminal diameter was 2.342 mm proximally and decreased serially distally with a reciprocal increase in intimal thickness, as we moved down towards the wrist. The IMT ratios in the proximal, mid, and distal segments were 0.530, 0.584, and 0.501, respectively (Ref.  Table 2). Abnormal Intimal thickening was found in 10 patients.

### 3.3. Intraoperative Morphometry

Radial artery was thick on palpation in 6 patients and intraoperative morphometric assessments revealed a mean arterial wall thickness of 0.502 mm and 0.548 mm at the proximal and distal ends of the harvested radial arteries, respectively. The respective proximal and distal arterial diameters were 2.30 mm and 2.18 mm.

### 3.4. Postoperative Histopathology Based Morphometry

The findings of postoperative morphometry studies showed similar trends as preoperative morphometry (Table 3).

We observed that, as far as dimensions and measurements of RA are concerned, there was good correlation among the various modalities.

### 3.5. Correlation between Findings of Preoperative USG and Postoperative Morphometry

**Figure 1:**
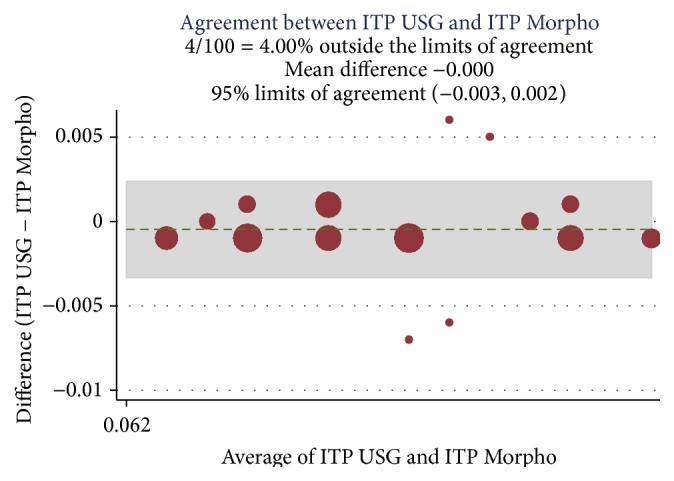
Intraclass Correlation Coefficient (ICC) was 0.91 with mean difference of −0.001; limit of agreement was 95% with upper and lower limits of −0.003 and 0.002. 4% values are above the limit of agreement, which means both of these methods are in agreement.

**Figure 2:**
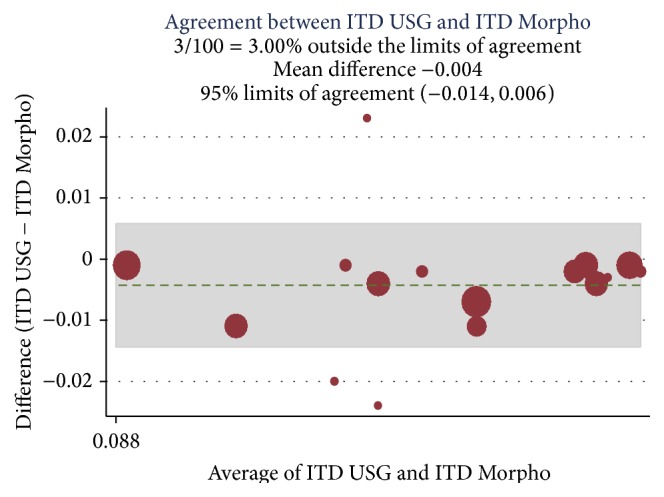
ICC was 0.90 with mean difference of −0.004; limit of agreement was 95% with upper and lower limits of −0.014 and 0.006. 3% values are above the limit of agreement, which means both methods are in agreement.

**Figure 3:**
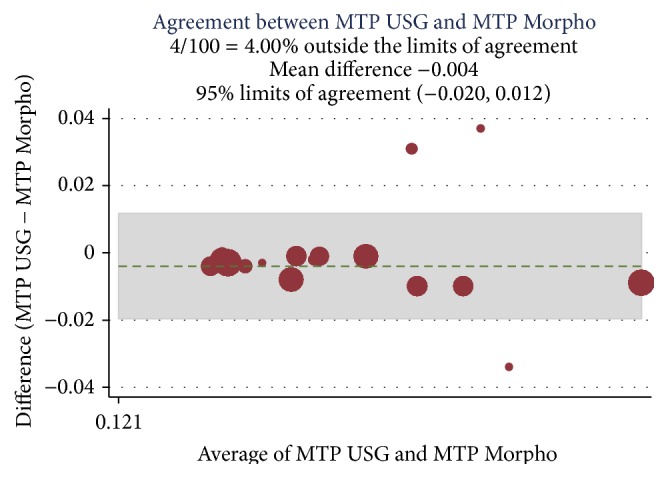
ICC was 0.91 with mean difference of −0.004; limit of agreement was 95% with upper and lower limit of −0.020 and 0.012. 4% values are above the limit of agreement, which means both of the methods are in agreement.

**Figure 4:**
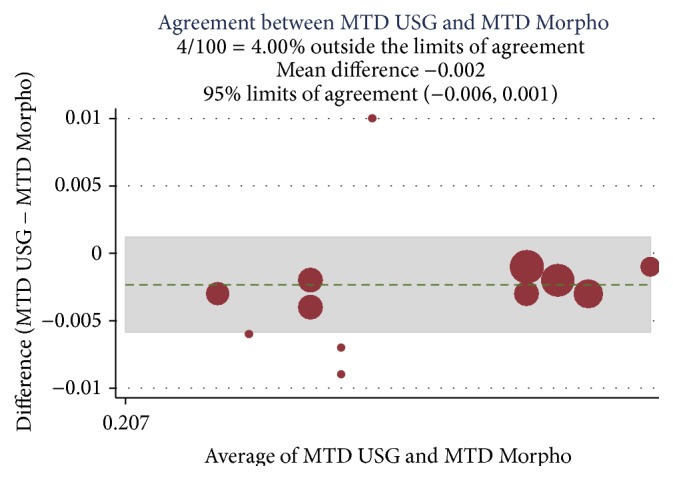
ICC was 0.89 with mean difference of −0.002; limit of agreement was 95% with upper and lower limits of −0.006 and 0.001. 4% values are above the limit of agreement, which means both of these methods are in agreement.

**Figure 5:**
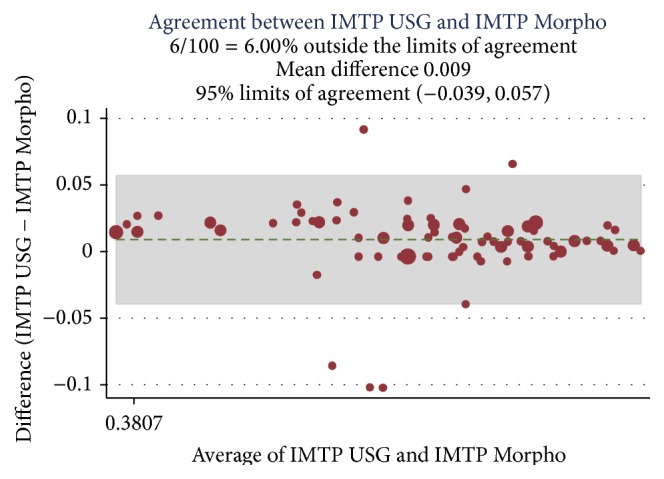
ICC was 0.89 with mean difference of −0.009; limit of agreement was 95% with upper and lower limits of −0.039 and 0.057. 6% values are above the limit of agreement, which means both of these methods are in agreement.

**Figure 6:**
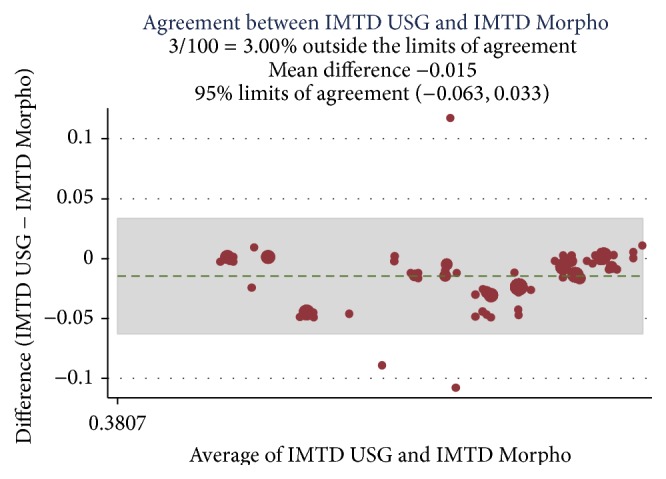
ICC was 0.92 with mean difference of −0.015; limit of agreement was 95% with upper and lower limits of −0.063 and 0.033. 3% values are above the limit of agreement, which means both of these methods are in agreement.

A correlation was established among the preoperative Doppler USG and postoperative histopathology assessment and morphometric findings (Ref.  Table 4) and Bland Altman analysis plotted for intimal thickness (Ref. Figures 1 and 2), medial thickness (Ref. Figures 3 and 4), and intima-media thickness ratio (Figures 5 and 6).

We observed that as far as dimensions and measurements of radial artery are concerned, there was good correlation among the various modalities.

## 4. Discussion

RA is now well established as the best arterial conduit after the internal mammary artery and there exists strong and consistent evidence of the superior long-term patency of RA over the saphenous vein. There is also rapidly growing body of evidence that this superior patency of RA translates into improved clinical outcomes including mortality benefits [1, 2]. Indeed, the current authors recently published a study from the same cohort of patients showing that clinical profile of the patients was not a precluding factor in the use of this conduit [3]. However, it has also been seen that the RA has a significantly greater prevalence of intimal hyperplasia and atherosclerosis, reported variably from 5.3% [4] to 31.5% [5]. Further, there appears to be a difference in functional characteristics between the proximal and distal ends of RA with the proximal segment demonstrating more vasoreactivity and force of contraction in response to vasopressors [6].

Doppler has been validated against histological measurements for providing reliable data on luminal diameter and intima-media thickness of carotid arteries [7–9]. Doppler USG also has been used to assess the hand collateral circulation and to validate Allen's test [10]. However, though several authors have used preoperative USG and intraoperative morphometry for deciding the suitability of the use of the RA as a conduit in CABG, there is no data available validating the preoperative USG against postoperative histopathology examination and morphometry for radial arteries. In recent years, the possibility of measuring RA vessel wall abnormalities and measuring the intimal-medial thickness has gained interest. Kim et al. used high resolution USG for measuring the RA wall thickness (intima-media thickness) in haemodialysis patients and validated it with histology-based measurements on samples obtained during AV fistula creation at wrist (*r* = 0.800, *p* < 0.001) [11]. However, we could not locate a single paper in published English language literature comparing and validating preoperative RA ultrasonography against histopathology for use of RA as a conduit for CABG. To the best of our knowledge, this is the first paper on the subject. In our study, we found that the preoperative Doppler ultrasound, intraoperative and postoperative histopathology based morphometric measurements of intima-media thickness ratio, intimal hyperplasia, and luminal diameter showed good correlation and, when indicated, preoperative Doppler ultrasound could provide useful and reliable data on suitability of RA for its use as a conduit in CABG.

We found that the proximal ends of RA had a greater luminal diameter and less thicker wall than the distal ends. As we proceed distally, the wall thickness due to increased thickness of both intima and media, with the latter having a greater contribution, led to decreased IMT ratio compared to proximal end. This was also the finding of Bhan et al. [12] who found that the prevalence of atherosclerotic disease was higher at the distal end with comparative morphometric analysis revealing significantly smaller percentage of luminal narrowing, intimal thickness index, and intima to media ratios in the proximal segments compared with the distal segments (*p* > 0.001). This has practical connotation, as the distal end of the RA is grafted to the coronary and at the time of doing the anastomosis to the aorta; the extra length of the RA conduit is excised and discarded which obviously comes from the proximal end of RA, which in fact is the better part. It is therefore suggested that near accurate length of the graft should be decided before the distal anastomosis and if any part of the conduit has to be discarded, it should be the distal and not the proximal end. On the contrary, Ueyama et al. [13], expressing the pathological index of arteriosclerosis as a ratio (internal luminal area/tunica media area), found no significant difference between the mean ratio of the proximal (0.177 ± 0.033) and the distal (0.258 ± 0.132) ends of the RAs. Although this did not achieve statistical significance, apparent trends are towards a higher degree of atherosclerosis in the distal end. Further, the proximal end was larger than the distal [13], thereby lending credence to our contention that the proximal segment of the RA should be preferred to the distal.

A confounding factor in this study is that the histological confirmation of Doppler findings was only possible in distal and proximal segments.

## 5. Conclusions

Doppler USG is an accurate screening test for evaluation of the suitability of RA, in terms of intimal/medial thickness and luminal diameter as a conduit in CABG, and has been validated by both morphometric and histopathology based studies. However, that does not imply that a case can be made out for routine Doppler screening of all radial arteries prior to CABG [14]. Hence, preoperative Doppler scanning may be considered in cases with palpable radial disease, those with widespread peripheral vascular disease, or cases with a positive Allen's test where one is under pressure to use as many arterial grafts as possible [14].

## Figures and Tables

**Table 1:** Patient demographics.

Number of patients	100
Males : females	79 : 21
Mean age (years)	61.45
Mean Body Surface Area (m^2^)	1.74
Mean left ventricular ejection fraction (%)	51.57
Double vessel disease	17
Triple vessel disease	83
Diabetes mellitus	54
Hypertension	69
Dyslipidemia	10
Smoking	33
Peripheral vascular disease	6
Stroke	3

**Table 2:** Preoperative radial Doppler measurements; (Dia: diameter, D: distal, IMTR: intima-media thickness ratio, ITL intimal thickening, Lum: luminal, M: mid, MT: medial thickening, and P: proximal).

S. number	Findings	Mean value in mm (S. numbers 1–9)
1	Lum Dia (P)	2.342
2	Lum Dia (M)	2.264
3	Lum Dia (D)	2.164
4	IT (P)	0.064
5	IT (M)	0.075
6	IT (D)	0.1
7	MT (P)	0.137
8	MT (M)	0.174
9	MT (D)	0.211
10	IMTR (P)	0.530
11	IMTR (M)	0.584
12	IMTR (D)	0.501

**Table 3:** Postoperative histopathology.

S. number	Findings in mm (S. numbers 1, 2, and 4)	Proximal	Distal
1	Intimal thickness	0.065	0.105
2	Medial thickness	0.142	0.213
3	Intima-media thickness ratio	0.534	0.501
4	Luminal diameter	2.351	2.165

**Table 4:** Comparison of USG and histology based morphometry; IMTRD: intima-media thickness ratio distal, IMTR: intima-media thickness ratio proximal, ITD: intimal thickness distal, ITP: intimal thickness proximal, MTD: medial thickness distal, and MTP: medial thickness proximal.

	Intraclass correlation coefficient (ICC)	Mean difference	Limit of agreement (upper and lower limit with 95% limits of agreement)
USG ITP and morpho-ITP	0.91	−0.001	−0.003 to 0.002
USG ITD and morpho-ITD	0.90	−0.004	−0.014 to 0.006
USG MTP and morpho-MTP	0.91	−0.004	−0.020 to 0.012
USG MTD and morpho-MTD	0.89	−0.002	−0.006 to 0.001
USG IMTRP and morpho-IMTRP	0.89	0.009	−0.039 to 0.057
USG IMTRD and morpho-IMTRD	0.92	−0.015	−0.063 to 0.033
